# Nonsteroidal anti-inflammatory drugs sensitize epithelial cells to *Clostridioides difficile* toxin–mediated mitochondrial damage

**DOI:** 10.1126/sciadv.adh5552

**Published:** 2023-07-19

**Authors:** Joshua Soto Ocaña, Nile U. Bayard, Jessica L. Hart, Audrey K. Thomas, Emma E. Furth, D. Borden Lacy, David M. Aronoff, Joseph P. Zackular

**Affiliations:** ^1^ Division of Protective Immunity, Children's Hospital of Philadelphia, Philadelphia, PA 19104, USA.; ^2^ Department of Pathology and Laboratory Medicine, Perelman School of Medicine, University of Pennsylvania, Philadelphia, PA 19104, USA.; ^3^ Department of Pathology, Microbiology, and Immunology, Vanderbilt University Medical Center, Nashville, TN 37232, USA.; ^4^ Department of Medicine, Indiana University School of Medicine, Indianapolis, IN 46202, USA.; ^5^ Institute for Immunology, Perelman School of Medicine, University of Pennsylvania, Philadelphia, PA 19104, USA.

## Abstract

*Clostridioides difficile* damages the colonic mucosa through the action of two potent exotoxins. Factors shaping *C. difficile* pathogenesis are incompletely understood but are likely due to the ecological factors in the gastrointestinal ecosystem, mucosal immune responses, and environmental factors. Little is known about the role of pharmaceutical drugs during *C. difficile* infection (CDI), but recent studies have demonstrated that nonsteroidal anti-inflammatory drugs (NSAIDs) worsen CDI. The mechanism underlying this phenomenon remains unclear. Here, we show that NSAIDs exacerbate CDI by disrupting colonic epithelial cells (CECs) and sensitizing cells to *C. difficile* toxin–mediated damage independent of their canonical role of inhibiting cyclooxygenase (COX) enzymes. Notably, we find that NSAIDs and *C. difficile* toxins target the mitochondria of CECs and enhance *C. difficile* toxin–mediated damage. Our results demonstrate that NSAIDs exacerbate CDI by synergizing with *C. difficile* toxins to damage host cell mitochondria. Together, this work highlights a role for NSAIDs in exacerbating microbial infection in the colon.

## Teaser

Off-target effects of NSAIDs enhance damage done by the important nosocomial pathogen, *C. difficile*.

## INTRODUCTION

During colonization, enteric pathogens are exposed to a complex ecosystem and numerous factors influence infection outcomes (*1*–*3*). External pressures shaping pathogen behavior include the host immune landscape, the gut microbiota, dietary factors, and xenobiotics. Understanding pathogenic behavior in the context of these immune, ecological, and environmental factors is essential to understanding mechanisms of disease manifestation and developing preventative and therapeutic strategies for this growing problem. One of the most important enteric pathogens is *Clostridioides difficile.* This nosocomial pathogen is the leading cause of antibiotic-associated diarrhea worldwide and an urgent public health threat (*4*). Infection by *C. difficile* leads to a wide range of disease manifestations that vary in severity from mild diarrhea to complex infection and death (*5*). The factors that influence this wide spectrum of clinical outcomes remain largely unclear. Emerging evidence suggests that previously unappreciated environmental factors, such as diet and pharmaceutical drugs, influence susceptibility to infection and disease manifestation (*6*–*10*). However, we still know very little about the influence of xenobiotics and pharmaceutical drugs on *C. difficile* infection (CDI).

Nonsteroidal anti-inflammatory drugs (NSAIDs) are the most prescribed drugs worldwide and are widely used to treat pain and reduce inflammation (*11*). These drugs inhibit cyclooxygenase (COX) enzymes 1 and 2 leading to a decreased production of the lipid mediators prostaglandins, prostacyclin, and thromboxane. Despite their important role in pain and inflammation management, NSAID use can lead to gastrointestinal (GI) complications and dysregulation of inflammatory responses (*12*, *13*). Long-term NSAID use is associated with intestinal injury and stomach ulcers (*14*–*16*). In the small intestine, these drugs cause numerous enteropathies, including bleeding and perforation of the intestinal tissue (*17*, *18*). It is hypothesized that NSAID-mediated enteropathy in the upper GI tract is driven by mucosal function impairment via COX enzyme inhibition (*12*). COX inhibition leads to decreased production of prostaglandins and prostacyclin stunting the cytoprotective effects these molecules exert in the GI tract. Beyond COX inhibition, NSAIDs are known to have off-target effects that can disrupt mitochondrial functions by uncoupling epithelial oxidative phosphorylation in the small intestine (*19*). This mechanism is dependent on the drug’s acidity, which allows acidic NSAIDs to partition into the mitochondrial membrane and ultimately disrupt mitochondrial functions (*20*). To date, the impact of NSAID-mediated mitochondrial effects on the development of enteropathy independent of COX enzyme inhibition has not been defined. Moreover, little is known about the role of off-target effects of NSAIDs in the colon, and the role of these effects on infection of the colon has not been explored in-depth.

NSAID usage is a risk factor for patients with inflammatory bowel disease (IBD) and is associated with spontaneous flares of inflammation in high-risk patients (*21*, *22*). NSAIDs are also associated with an increased risk for CDI and disease, and it is well established that NSAID treatment should be avoided in patients suspected of having CDI (*23*–*26*). Notably, our previous work established that prior exposure to the NSAID, indomethacin, can exacerbate CDI and markedly increase mortality in a mouse model of infection (*26*, *27*). Treatment with NSAIDs leads to dysregulation of the immune response, delocalization of tight junction proteins in colonic epithelial cells (CECs), and disruption of the microbiome during CDI. Together, this work demonstrated that indomethacin exposure, even days before CDI, can have a long-lasting and marked impact on the outcomes of CDI in an animal model. However, the mechanism by which NSAID preexposure sensitizes mice to severe CDI, and the mechanism associated with NSAID-mediated alteration to the epithelium is unknown. In our study, we sought to define the molecular mechanism of NSAID-induced mortality during CDI and elucidate the interactions between NSAIDs, *C. difficile,* and the colonic epithelium during infection. To our surprise, we did not find a primary role for COX inhibition in NSAID-mediated disease enhancement in CECs. Instead, we show that NSAIDs exacerbate *C. difficile*–associated disease by sensitizing CECs to the effects of *C. difficile* toxins through off-target and synergistic effects on host mitochondria in the colonic epithelium. Disruption of mitochondrial functions increases CEC damage and inflammatory death and likely accounts for the established negative effects of NSAIDs on CDI. Together, this work defines the mechanism of NSAIDs on an important infection and describes an underappreciated off-target impact of indomethacin on the mitochondria of the colonic epithelium.

## RESULTS

### NSAIDs increase epithelial cell damage in an in vitro model of *C. difficile* intoxication

**Fig. 1.**
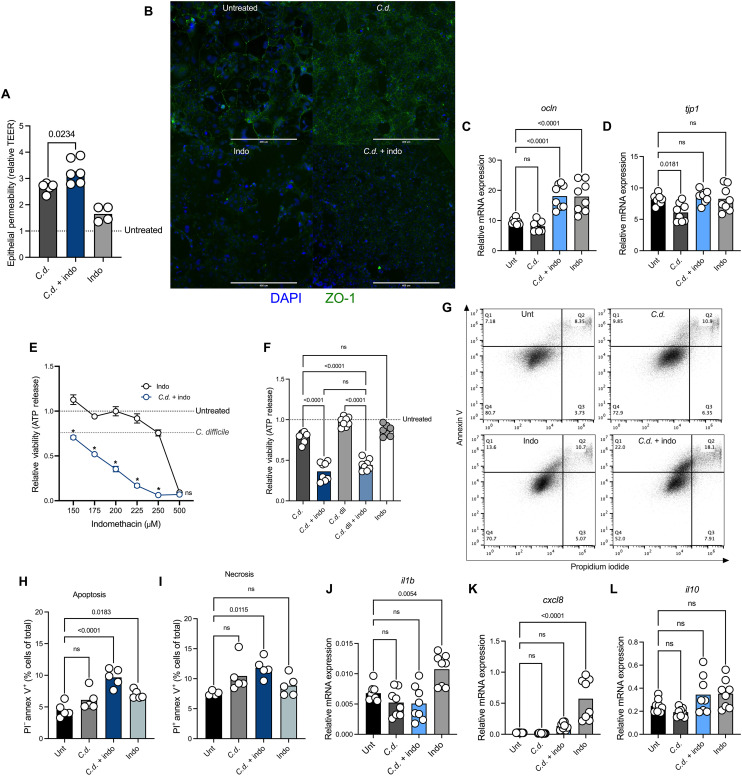
NSAIDs increase epithelial cell damage in an in vitro model of *C. difficile* intoxication. (**A**) Transepithelial electrical resistance (TEER) measured 6 hours after TcdB (*C.d.*) intoxication in differentiated Caco-2 cells after 16-hour treatment with indomethacin (indo) or vehicle control. “Untreated” (Unt) line represents baseline permeability of vehicle-treated cells [mean, *n* = 5 per treatment, one-way analysis of variance (ANOVA) with multiple comparisons]. (**B**) Representative ICC images of Caco-2 cells treated with TcdB (*C.d.*), indomethacin (indo), *C.d.* + indo, or vehicle control (Unt) and stained with an anti–ZO-1 antibody and DAPI. (**C** and **D**) Real-time rt-qPCR data for tight junction proteins *tjp1* and *ocln* (mean, *n* = 8 per treatment, one-way ANOVA with multiple comparisons). (**E**) Epithelial cell viability after treatment with increasing concentrations of indo, *C. difficile* supernatants at a 1:5 dilution (*C.d.*), *C.d.* + indo, vehicle control or mock-infected. Data normalized to mock-infected and vehicle control–treated cells (untreated line). Amount of cell death induced by *C. difficile* in gray line (mean ± SEM, *n* = 4 per indomethacin concentration, two-way ANOVA with multiple comparisons, *P < 0.0001). (**F**) Epithelial cell viability measured after treatment with 200 μM indo , *C.d.* supernatants at a 1:5 dilution, *C.d.* + indo, *C.d.* supernatants at a 1:15 dilution (*C.d.* dil), *C.d.* dil + indo, vehicle control or mock-infected. (mean, *n* = 8 per treatment, one-way ANOVA with multiple comparisons). Flow cytometry analyses of Caco-2 cells treated with TcdB (*C.d.*), indo, *C.d.* + indo, or vehicle control (Unt) and stained with an annexin V antibody and propidium iodide. (**G**) Representative flow plots. (**H**) Apoptotic cells gated on annexin V^+^ propidium iodide^−^ cells. (**I**) Necrotic cells gated on annexin V^+^, propidium iodide^+^ cells (mean, *n* = 5 per treatment, one-way ANOVA with multiple comparisons). (**J** to **L**) rt-qPCR in Caco-2 cells for the inflammatory markers *ilb, cxcl8,* and *il10*. (mean, *n* = 8 per treatment, one-way ANOVA with multiple comparisons).

Our previous work demonstrated that indomethacin worsens *C. difficile* disease, but the mechanisms of disease exacerbation remain unclear (*27*). Following colonization of the colon, *C. difficile* produces two potent exotoxins, TcdA and TcdB. These toxins are glucosyltransferases that trigger disassociation of the actin cytoskeleton, loss of tight junction integrity, and programmed cell death in CECs. Thus, to begin to define the molecular mechanisms of NSAID-mediated disease enhancement, we used an in vitro model of *C. difficile* intoxication of Caco-2 CECs. Indomethacin, a nonselective COX inhibitor that is known to cause enteropathy in the small intestine, was used as a representative NSAID in our studies (*28*). Using this Caco-2 cell epithelial barrier model, we observed that both indomethacin and recombinant TcdB treatment increased epithelial cell barrier permeability (Fig 1A). This effect was additive, as the combined effect of both TcdB and indomethacin was increased compared to each independent treatment. To determine whether this phenomenon was associated with tight junction integrity, as previously reported (*27*), we examined tight junction protein localization via immunocytochemistry (ICC) and expression via real-time quantitative polymerase chain reaction (rt-qPCR). Following treatment with indomethacin and/or *C. difficile* toxin, we observed changes in cell morphology that corresponded with changes in zona occludens-1 (ZO-1) (Fig. 1B). Exposure of cells to TcdB was characterized by increased formation of puncta in cell borders, whereas both indomethacin alone and the combination indomethacin and TcdB treatment decreased the amount of protein localizing between cells. We did not observe changes in expression of ZO-1 (*tjp1*) after cotreatment with indomethacin and TcdB (Fig. 1C). However, treatment with indomethacin and the combination of TcdB and indomethacin increased expression of the tight junction protein occludin (*ocln*) (Fig. 1D). These data suggest that indomethacin and TcdB both act on the colonic epithelium and that the combined effect of these two insults is the driver of diminished barrier function observed during infection.

*C. difficile* toxins induce cell death through multiple independent pathways in epithelial cells (*29*–*31*). To determine whether NSAIDs enhance or alter toxin-mediated cell death in CECs, we explored the impact of indomethacin on Caco-2 cell survival in our in vitro model. As expected, we observed that indomethacin, independent of *C. difficile*, can cause cell death in Caco-2 cells in a dose-dependent manner (Fig. 1E). However, we found that at concentrations of indomethacin that do not cause substantial cell death, addition of *C. difficile–*filtered supernatants, which contain both toxins (TcdA and TcdB), markedly enhanced cell death (Fig. 1E). This suggests that the combinatory effect of NSAID treatment and intoxication by *C. difficile* toxins is highly damaging to CECs. Notably, pretreatment of cells with indomethacin lowered the threshold of *C. difficile* supernatants required to increase CEC death, suggesting that indomethacin sensitizes cells to the effects of the toxins (Fig. 1F). The impact of NSAIDs on Caco-2 cell death was not limited to indomethacin, as other NSAIDs such as ibuprofen, naproxen, and aspirin also increased cell death in the presence of *C. difficile* toxins (fig. S1, A to C). *C. difficile* toxins have the capacity to induce multiple programmed cell death pathways in epithelial cells, a phenomenon that is toxin and concentration dependent (*32*). Thus, we investigated the mechanisms of induced cell death by gating populations of cells that were annexin V^+^ propidium iodide^−^ to identify apoptotic cells and annexin V^+^ propidium iodide^+^ for necrotic cells. Combinatory treatment with indomethacin and TcdB led to increased numbers of apoptotic cells, a phenomenon that was also mediated by indomethacin treatment alone (Fig. 1, G and H). We observed an increase in necrotic cell death following indomethacin and TcdB combinatory treatment, which was unique to this treatment group (Fig. 1, G and I). Last, we assessed the inflammatory profile of CECs after treatment with TcdB and indomethacin via rt-qPCR. We observed an increase in the expression of the inflammatory cytokines interleukin-1β (IL-1β) (*il1b*) and IL-8 (*cxcl8*) following treatment with indomethacin (Fig. 1, J and K) and did not observe changes in the anti-inflammatory cytokine IL-10 (Fig. 1L). This suggests that NSAID-mediated cell death during CDI is likely inflammatory. Together, these data demonstrate that indomethacin sensitizes CECs to *C. difficile* toxins, and the combination of NSAIDs and *C. difficile* toxins impairs colonic epithelial cell barrier functions by enhancing cell death, permeability, and inflammation.

### NSAIDs effects on CDI are independent of action on COX enzymes and prostaglandins

**Fig. 2.**
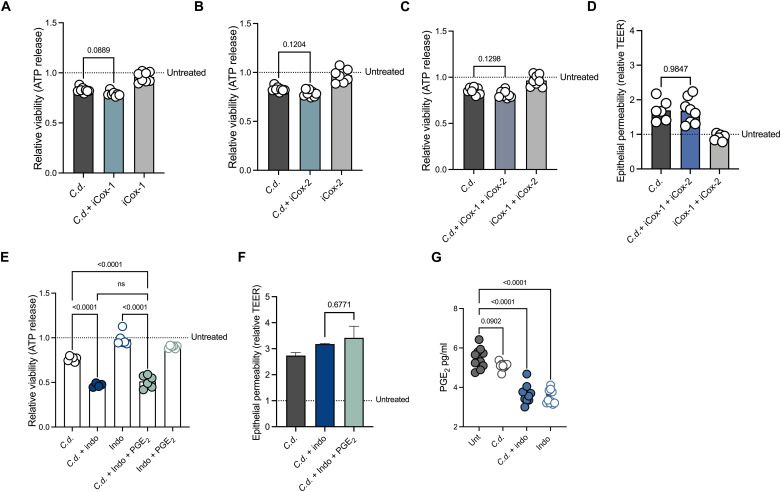
NSAIDs effects on CDI are independent of action on COX enzymes and prostaglandins. (**A** to **C**) Epithelial cell viability measured after treatment with valeroyl silyciate (iCox-1), celecoxib (iCox-2) combination of iCox-1 + iCox-2, *C. difficile* supernatants at a 1:5 dilution (*C.d.*), combination *C.d.* + iCox-1, *C.d.* + iCox-2, or *C.d.* + iCox-2 + iCox-2, vehicle control, or mock-infected. Data normalized to mock-infected and vehicle control–treated cells (untreated line) (mean, *n* = 8 per each single and double treatment, one-way ANOVA with multiple comparisons). (**D**) TEER measured 4 hours after TcdB (*C.d.*) intoxication in differentiated Caco-2 cells after 16-hour treatment with iCox-1 and iCox2 or vehicle control. Dotted line represents baseline permeability of vehicle-treated cells (mean, *n* = 6 per treatment, one-way ANOVA with multiple comparisons). (**E**) Epithelial cell viability measured after treatment with 200 μM indo, *C.d.* supernatants at a 1:5 dilution, combination *C.d.* + indo, indo + PGE_2_, combination of *C.d.* + indo + PGE_2_, vehicle control, or mock-infected. Data normalized to mock-infected and vehicle control–treated cells (untreated line) (mean, *n* = 6 per treatment, one-way ANOVA with multiple comparisons). (**F**) TEER measured at 6 hours after TcdB (*C.d.*) intoxication in differentiated Caco-2 cells after 16-hour treatment with indo, indo + PGE_2_ or vehicle control. Dotted line represents baseline permeability of vehicle-treated cells (mean, n = 3 per treatment, one-way ANOVA with multiple comparisons). (**G**) Concentrations of PGE_2_ in CECs after treatment with TcdB (*C.d.*), indo, combination *C.d.* + indo or vehicle control (Unt) (mean ± SEM, *n* = 10 per treatment, one-way ANOVA with multiple comparisons).

NSAIDs target COX enzymes and inhibit the production of prostaglandins, prostacyclin, and thromboxane. In the gut, prostaglandin E_2_ (PGE_2_) has been shown to be important during inflammation and infection (*33*, *34*). On the basis of this, we hypothesized that indomethacin-mediated inhibition of PGE_2_ contributes to enhanced *C. difficile*–associated disease. In support of this, we have previously demonstrated that treatment with the PGE_2_ analog, misoprostol, protected mice from severe CDI (*35*). Here, we sought to further define the contribution of COX enzyme inhibition and PGE_2_ production during CDI. First, we explored the impact of selective COX-1 and COX-2 inhibitors on intoxication with *C. difficile* toxin supernatants in our model epithelium. Unexpectedly, we were unable to demonstrate that COX-1 or COX-2 inhibition negatively affects epithelial cell survival during *C. difficile* intoxication (Fig. 2, A and B). Consistent with this, combination of COX-1 and COX-2 inhibitors did not affect CEC death in our in vitro system (Fig. 2C). We also measured epithelial cell permeability after cotreatment with selective COX-1 and COX-1 inhibitors and showed that consistent with the previous data, these did not enhance epithelial cell permeability after TcdB treatment (Fig. 2D). To test whether PGE_2_ supports epithelial cell health during CDI, we supplemented NSAID-treated CECs with PGE_2_ during *C. difficile* intoxication. The addition of PGE_2_ did not protect cells from enhanced permeability and cell death following combinatory treatment with indomethacin and *C. difficile* toxin (Fig. 2, E and F). To further strengthen this interpretation, we measured PGE_2_ levels after indomethacin treatment in our model epithelium. We observed a decrease in PGE_2_ levels after treatment with indomethacin and combination of indomethacin and TcdB (Fig. 2G). These data suggest that although inhibition of prostaglandin production occurs following indomethacin treatment, this is not the primary mode of NSAID-mediated effects on CECs during *C. difficile* intoxication. Furthermore, signaling through PGE_2_ receptors, although likely playing a role, is not alone sufficient to protect the epithelial cell barrier in these conditions. This strengthens the hypothesis that interrupting prostaglandin signaling through the inhibition of COX enzymes and prostaglandin signaling itself is not absolutely required for NSAIDs to induce damage to the colonic epithelium during CDI.

### NSAIDs exacerbate CDI independent of COX inhibition

**Fig. 3.**
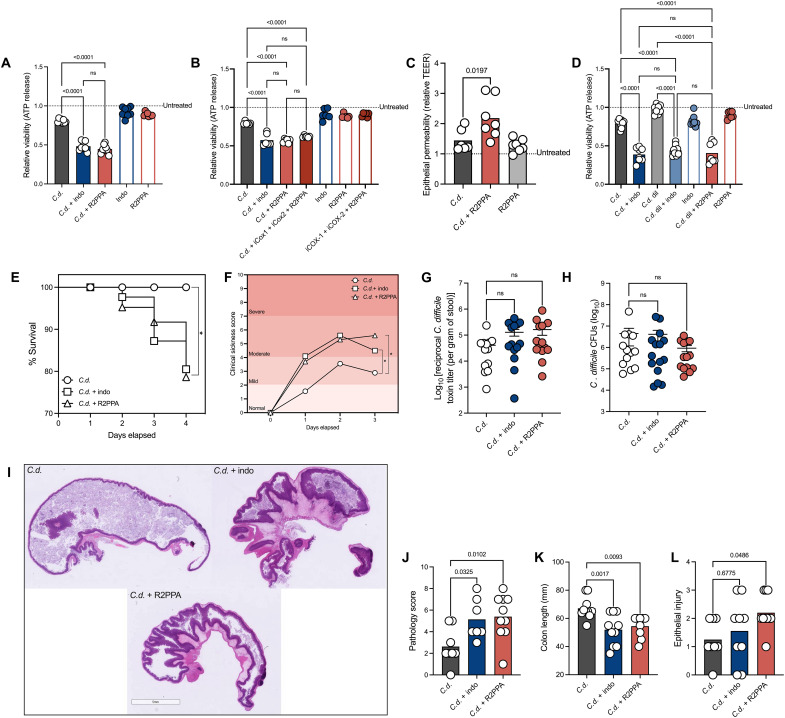
NSAIDs exacerbate CDI independent of COX inhibition. (**A** and **B**) Epithelial cell viability measured after treatment with 200 μM indo, R2PPA, combination of R2PPA + iCox1 + iCox2 and or vehicle control, followed by intoxication with *C. difficile* supernatants at 1:5 dilution (*C.d.*) or mock-infected. Data normalized to mock-infected and vehicle control–treated cells (untreated line) (mean, *n* = 8 per treatment, one-way ANOVA with multiple comparisons). (**C**) TEER measured 4 hours after TcdB (*C.d.*) intoxication in differentiated Caco-2 cells after treatment with R2PPA or vehicle control. Dotted line represents baseline permeability of vehicle-treated cells (mean, *n* = 6 per treatment, one-way ANOVA with multiple comparisons). (**D**) Epithelial cell viability measured after treatment with 200 μM indo, R2PPA, *C. difficile* supernatants at a 1:5 dilution (*C.d.*), *C. difficile* supernatants at a 1:15 dilution (*C.d.* dil) or combination of *C.d.* dil + indo or *C.d.* dil + R2PPA, vehicle control or mock-infected. Data normalized to mock-infected and vehicle control–treated cells (untreated line) (mean, *n* = 8 per treatment, one-way ANOVA with multiple comparisons). Mice pretreated with indo or R2PPA followed by infection with *C. difficile* Cd196 (*C.d*) were (**E** and **F**) monitored for survival, weight loss, behavior, and stool consistency (*n* = 15 mice per group, log-rank test). (**G** and **H**) *C. difficile* colony-forming units (CFUs) and toxin titers from stool of infected mice. Toxin titers measured by cytotoxicity and normalized to weight of stool (mean ± SEM, *n* = 15 mice per group, nonparametric one-way ANOVA with multiple comparisons). (**I**) Representative images of hematoxylin and eosin-stained tissues. (**J**) Colon length was measured (mean, *n* = 10 mice per group, one-way ANOVA with multiple comparisons). (**K** and **L**) Pathology scores from ceca of infected mice (mean, *n* = 8 for *C.d.*, *n* = 7 for *C.d.* + indo, and *n* = 10 for *C.d.* + R2PPA, one-way ANOVA with multiple comparisons). ns, not significant.

NSAIDs have been shown to have off-target effects independent of the COX enzymes (*36*). Specifically, NSAIDs can interact with mitochondria and have the potential to uncouple cellular mitochondrial functions. This is driven by the ability of NSAIDs to enter the inner mitochondrial membrane and form ionophores that allow hydrogen ions to enter the inner mitochondrial membrane (*20*). These mitochondrial interactions lead to subsequent drops in adenosine 5′-triphosphate (ATP) production, calcium release into the cytosol, and activation of programmed cell death pathways (*37*, *38*). To examine the role of off-target effects of NSAIDs in the context of *C. difficile* intoxication, we used *R*-(−)-2-phenylpropionic acid (R2PPA), an NSAID precursor that is structurally similar to an NSAID, lacks the capacity to inhibit COX enzyme, and has the ability to uncouple mitochondrial functions (*39*). We observed that R2PPA induced cell death in CECs at a similar level to indomethacin after intoxication with filtered supernatants from *C. difficile* cultures (Fig. 3A). The addition of a COX-1 and a COX-2 selective inhibitor to R2PPA did not further enhance cell death above R2PPA or indomethacin alone (Fig. 3B), demonstrating that COX enzyme inhibition is not required to induce epithelial cell damage during CDI. We further assessed the role of R2PPA on epithelial cell permeability and observed that like indomethacin, this molecule exacerbates epithelial permeability in the presence of TcdB (Fig. 3C). Notably, when cells were pretreated with R2PPA and treated with a concentration of *C. difficile* toxins that do not induce cell death, we saw enhanced sensitivity to *C. difficile* supernatants (Fig. 3D). These data further demonstrate that NSAIDs have a marked effect on toxin-mediated damage independent of COX activity. Next, to test the role of off-target effects during CDI, we infected mice following pretreatment with R2PPA and indomethacin. Mice infected with the ribotype 027 strain, Cd196, showed equal enhancement in disease severity and mortality when treated with either R2PPA or indomethacin compared to untreated control mice infected with *C. difficile* only (Fig. 3, E and F). Indomethacin and R2PPA-treated mice had similar *C. difficile* burdens and toxin titers in their stool compared to untreated *C. difficile*–infected mice indicating that NSAIDs do not affect pathogen behavior during CDI (Fig. 3, G and H). The in vivo impact of NSAIDs was not limited to indomethacin since we observed an increase in mortality and similar *C. difficile* burdens after pretreatment with the NSAID aspirin (fig. S2, A and B). Colonic tissues from the mice treated with R2PPA and indomethacin both exhibited higher pathology scores and shorter colon lengths compared to *C. difficile*–infected only mice (Fig. 3, I to K). Notably, R2PPA and indomethacin-treated mice showed higher epithelial cell injury compared to the *C. difficile–*only–infected mice supporting previous in vitro data showing that NSAIDs increase CEC damage during infection (Fig. 3L). Together, these data demonstrate that NSAIDs likely exacerbate CDI independent of their COX enzyme–inhibition activity.

### NSAIDs and *C. difficile* synergize to disrupt mitochondrial functions in CECs

**Fig. 4.**
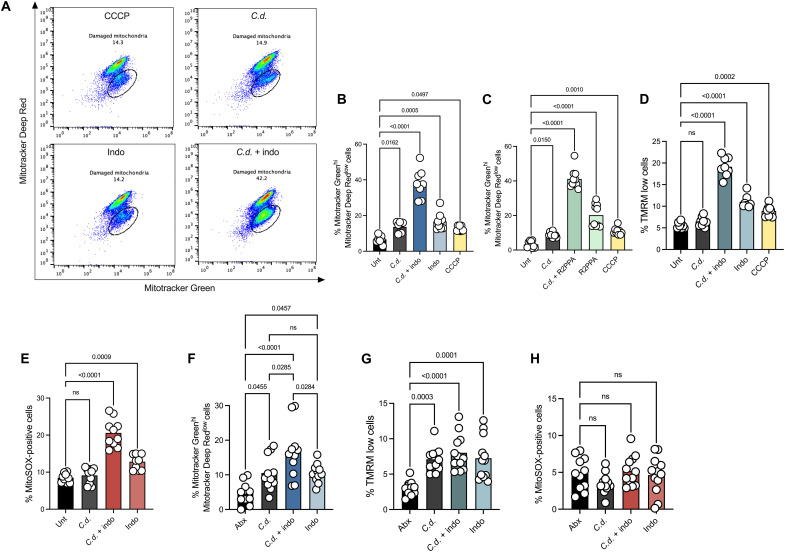
NSAIDs and *C. difficile* synergize to disrupt mitochondrial functions in CECs. Caco-2 cells treated with indo, TcdB (*C.d.*), combination of *C.d.* + indo, CCCP (positive control) or vehicle control (Unt) and stained with Mitotracker Green and Mitotracker Deep Red. (**A**) Representative flow plots. Circled population denotes the percentage of the population with damaged mitochondria by gating Mitotracker Green^hi^ and Mitotracker Deep Red^low^ cells. (**B**) Percentage of Mitotracker Green^hi^ and Mitotracker Deep Red^low^ cells plotted (mean, *n* = 8 per treatment, one-way ANOVA with multiple comparisons). (**C**) Percentage of Mitotracker Green^hi^ and Mitotracker Deep Red^low^ Caco-2 cells after treatment with R2PPA, TcdB (*C.d.*), combination of *C.d.* + R2PPA, CCCP, or vehicle control (Unt) (mean, *n* = 10 per treatment, one-way ANOVA with multiple comparisons). (**D**) Percentage of TMRM-low Caco-2 cells (mean, *n* = 10 per treatment, one-way ANOVA with multiple comparisons). (**E**) Percentage of MitoSOX-positive Caco-2 cells (mean, *n* = 10 per treatment, one-way ANOVA with multiple comparisons). (**F**) Percentage of Mitotracker Green^hi^ and Mitotracker Deep Red^low^ CECs isolated from mice after treatment and/or infection with cefoperazone (Abx), indo, *C. difficile* Cd196 (*C.d.*) or *C.d.* + indo (mean, *n* = 12 per treatment, one-way ANOVA with multiple comparisons). (**G**) Percentage of TMRM-low CECs isolated from infected mice (mean, *n* = 12 per treatment, one-way ANOVA with multiple comparisons). (**H**) Percentage of MitoSOX-positive CECs cells isolated from infected mice (mean, *n* = 12 per treatment, one-way ANOVA with multiple comparisons).

Mitochondria are cell organelles that control many cellular functions including metabolism and cell death (*40*, *41*). Evidence shows the ability of NSAIDs to interact with and uncouple mitochondria, ultimately disrupting cellular functions. Studies have also described a potential role for mitochondria during intoxication with *C. difficile* toxins in epithelial cells (*42*, *43*). Thus, we postulated that NSAIDs may be sensitizing CECs to *C. difficile* toxins via effects on mitochondria. To specifically test the role of mitochondrial uncoupling in NSAID-mediated damage during CDI, we used flow cytometry to assess mitochondrial functions. When Caco-2 cells were treated with TcdB and indomethacin, we observed an increase in damaged mitochondria compared to both *C. difficile* or indomethacin only groups, measured by gating populations of cells that were Mitotracker Green^hi^ and Mitotracker Deep Red^low^ (Fig. 4, A and B) (*44*). Similar to indomethacin treatment, we observed that combination of TcdB and R2PPA increased the number of Mitotracker Green^hi^ and Mitotracker Deep Red^low^ cells (Fig. 4C). These data demonstrate that *C. difficile* toxin has the potential to cause mitochondrial damage, and the mitochondrial uncoupling capacity of NSAIDs drives epithelial damage during CDI. To further characterize mitochondrial functions in vitro, we measured mitochondrial membrane potential by gating tetramethylrhodamine methyl ester (TMRN) low cells. From this, we observed that the combination of indomethacin and TcdB increased the percentage of cells with decreased mitochondrial membrane potential when compared to single treatments (Fig. 4D). Next, we assessed the production of mitochondrial superoxide during NSAID and TcdB treatment using MitoSOX. We observed that the combination of TcdB and indomethacin increased the percentage of MitoSOX-positive cells indicating an increase in superoxide production by CECs (Fig. 4E). These data demonstrate that NSAIDs and *C. difficile* toxin synergize and their induced damage to the colonic epithelium is in part due to their ability to disrupt mitochondrial function during infection.

To define the synergistic effect of NSAIDs and *C. difficile* toxins in the context of infection, we next measured mitochondrial function in our model of CDI. Mice pretreated with indomethacin and subsequently infected with *C. difficile* showed increased levels of CECs with damaged mitochondria compared to cells from mice that were treated with indomethacin, *C. difficile* or cefoperazone only (Fig. 4F). Moreover, we observed that *C. difficile,* indomethacin, or both in combination equally decreased mitochondrial membrane potential in murine CECs, as measured by TMRN low cells (Fig. 4G). Our data demonstrate that during infection in mice, *C. difficile* impairs mitochondrial functions in CECs independent of NSAIDs. This effect is amplified in the context of NSAID treatment. Inconsistent with our in vitro studies, when colonic epithelial mitochondrial superoxide production was measured in vivo, we did not observe any changes between treatment groups, suggesting that our murine model of infection may not be sensitive enough to detect this effect (Fig. 4H). Together, these data demonstrate that NSAIDs enhance *C. difficile*–associated disease by perturbing mitochondrial functions, leading to epithelial cell damage during infection. These findings provide an unexpected framework for how NSAIDs may worsen the clinical outcomes of this important nosocomial infection and provide important insights into the off-target effects of NSAIDs on the colonic epithelium.

## DISCUSSION

NSAIDs are detrimental during CDI; however, the mechanism of NSAID-mediated disease enhancement has been unclear. Here, we further define mechanisms of NSAID-mediated enhancement of disease during CDI and uncovered unexpected off-target effects of NSAIDs in the colon that drive epithelial damage during infection. We demonstrate that the effects of NSAIDs in the colon are independent of the canonical targets of these drugs during CDI. Instead, we propose that NSAIDs are affecting mitochondria in CECs leading to increased sensitivity to *C. difficile* toxins and increased mortality during CDI. These results highlight an underappreciated role of NSAIDs during GI infection in the colon.

It is appreciated that NSAID-induced damage to the GI epithelium is mediated by the action of COX-1 and COX-2 inhibition. Historic data have also highlighted the off-target effects these drugs have on host cells and their ability to act as mitochondrial uncoupling agents in the small intestine. However, the link between the off-target effects of NSAID and damage has not been defined in the colon during infection with an enteric pathogen. Moreover, in vitro studies have shown the potential of *C. difficile* toxins to dysregulate CEC mitochondrial functions. In this study, we demonstrate during an infection setting that the *C. difficile* toxins perturb mitochondrial functions in CECs, and consistent with previous studies, our data point to the substantial role mitochondrial functions play during *C. difficile* pathogenesis. Our work further suggests that NSAIDs perturb the colonic epithelium leading to epithelial cell death and permeability independent of COX enzyme inhibition. In the context of CDI, NSAID pretreatment leads to enhancement of epithelial cell damage and sensitization of epithelial cells to *C. difficile* toxins. We postulate that this phenomenon is mediated by off-target effects of NSAIDs on CEC mitochondria, which sensitizes cells to the effects of *C. difficile* toxins leading to increased cell death, intestinal permeability, and inflammation. NSAIDs use, specifically the use of aspirin, is associated with Raye syndrome—a systemic disorder caused by abrupt insults to the mitochondria (*45*). It is also common practice to avoid providing NSAIDs to patients with CDI, but the underlying mechanisms behind this practice have not been well described. Our work sheds light on the clinical importance of NSAIDs in patients with CDI and why the combination of these two may be so detrimental. This study highlights the importance of the colonic epithelium during CDI and the essential role it plays in protecting the host from pathogenic bacteria. Our findings could be a starting point for further research that aims to understand the impact of mitochondrial functions during CDI. Furthermore, these data can inform how NSAID-mediated mitochondrial uncoupling affects other diseases such as small intestinal injury, IBD, and colorectal cancer.

## MATERIALS AND METHODS

### Bacterial strains and growth

*C. difficile* strain VPI10463 was used in this study and grown at 37°C in an anaerobic chamber (85% nitrogen, 10% hydrogen, and 5% carbon dioxide, Coy Laboratory Products) in brain heart infusion (BHI) broth (BD Life Sciences) supplemented with 0.5% yeast extract (BD Life Sciences) and 0.1% cysteine (Sigma-Aldrich) as previously described (*6*, *46*, *47*). For in vitro intoxication assays, VPI10463 cultures were grown to an optical density at 600 nm (OD_600_) of 0.7. Recombinant toxin was isolated from *C. difficile* strain VPI10463, and methods for purification are detailed below.

### Recombinant toxin

Recombinant TcdB was expressed and purified as previously described (*48*). Briefly, a plasmid encoding His-tagged TcdB (pBL377) was transformed into *Bacillus megaterium* according to the manufacturer’s protocol (MoBiTec). Six liters of Luria-Bertani medium supplemented with tetracycline (10 mg/liter) were inoculated with an overnight culture to an OD_600_ of ∼0.1. Cells were grown at 37°C and 220 rpm. Expression was induced with 5 g/liter of *d*-xylose once cells reached an OD_600_ of 0.3 to 0.5. After 4 hours, the cells were centrifuged and resuspended in 20 mM tris (pH 8.0), 500 mM NaCl, and protease inhibitors. An EmulsiFlex C3 microfluidizer (Avestin) was used at 15,000 lb/in^2^ twice to generate lysates. Lysates were then centrifuged at 40,000*g* for 20 min. Supernatant containing toxin was passed through a Ni-affinity column (HisTrap FastFlow Crude, GE Healthcare) initially. Further purification was performed using Q-Sepharose anion-exchange chromatography (GE Healthcare) and gel filtration chromatography in 20 mM Hepes (pH 6.9)–50 mM NaCl.

### Colonic cell lines

Caco-2 cells were provided by K. Hamilton (Children’s Hospital of Philadelphia) and were maintained in modified Eagle’s medium (MEM) supplemented with 10% fetal bovine serum (FBS), 1% GlutaMAX, 1% sodium pyruvate, and 1% nonessential amino acids. All cells were grown in a humidified incubator at 37°C with 5% CO_2_ atmosphere.

### Animal model of CDI

All mouse procedures were approved by the Children’s Hospital of Philadelphia Institutional Animal Care and Use Committee. Mice were obtained from The Jackson Laboratory and were C57BL6/J males that were 4 weeks of age at arrival. Mice were acclimated for 7 days, and bedding was mixed upon arrival. After acclimation, mice were given cefoperazone at 0.5 g/liter in the drinking water for 5 days, with changes in antibiotic water every 2 days. After 5 days, water was changed to untreated water.

After the 5-day antibiotic treatment, mice were orally gavaged with indomethacin (Cayman) at 10 mg/kg, aspirin (Cayman) at 10 mg/kg, or R2PPA (Sigma-Aldrich) at 100 mg/kg of body weight or vehicle phosphate-buffered saline (PBS) for two consecutive days. Then, mice were infected with 10^5^ spores of *C. difficile* ribotype Cd196. Mice were monitored for survival and were euthanized after reaching a terminal endpoint of appearing moribund or experiencing weight loss >20% from baseline. *C. difficile* colony-forming units were quantified daily from fecal samples. Samples were diluted and homogenized in PBS, serially diluted, and plated onto taurocholate cycloserine cefoxitin fructose agar. All animals were confirmed *C. difficile* culture negative upon arrival to the animal facility and on the day of infection.

### *C. difficile* toxin titers from feces

For quantification of *C. difficile* toxin titers via cytotoxicity assay, a Vero cell–rounding cytotoxicity assay was used. Briefly, Vero cells were plated at 1 × 10^4^ cells per well in a 96-well flat-bottom plate and incubated for 24 hours. Fresh fecal samples were homogenized in 1 ml of sterile PBS and pelleted at 4000*g* for 5 min. The supernatants were filtered through a 0.2-μm filter and titrated in 10-fold dilutions within the wells to a maximum dilution of 10^−8^. After overnight incubation, Vero cell rounding was assessed under ×10 magnification. Cytotoxicity data are expressed as the reciprocal value of the highest dilution that rounded 100% of the cells per gram of sample.

### Histological analysis

At necropsy, ceca were harvested, fixed in 10% formalin solution, and embedded in paraffin. Sections were stained with hematoxylin and eosin. Each section was assigned a disease score through blinded assessment by a pathologist based on previously described criteria. Histological scores were reported as a cumulative score of three independent scoring criteria: inflammation, edema, and epithelial cell damage unless otherwise noted.

### Cell survival assay

Caco-2 cells were plated in black 96-well plates at 3 × 10^4^ cells per well and incubated for 48 hours. After 48 hours, cell culture medium was refreshed, and cells were treated with indomethacin, ibuprofen (Cayman), naproxen (Cayman), aspirin, R2PPA, valeroyl silyciate (Cayman), celecoxib (Cayman), PGE_2_ (Cayman), or vehicle control [dimethyl sulfoxide (DMSO)] for 16 hours overnight. The next day, cells were intoxicated with filter-sterilized *C. difficile* VPI10463 cultures at a final dilution of 1:5 or 1:15 or mock-infected (BHI) and incubated for 8 hours. Cell viability was indicated by measuring ATP levels using the CellTiter Glo Reagent (Promega). Data were normalized to ATP levels of vehicle-treated and mock-infected cells.

### Permeability assays

Caco-2 cells were plated in 12-well insert plates (Corning) at 1.5 × 10^5^ cells and cultured for at least 14 days refreshing media every other day until resistance was >1000 ohms. Cells were treated with 500 μM indomethacin, 4 mM R2PPA, 10 μM valeroyl silyciate, 10 μM celecoxib, 1 mM PGE_2_, or vehicle control (DMSO) for 16 hours overnight. The next day, cells were intoxicated with recombinant TcdB at a concentration of 50 nM. Resistance was measured at 4 or 6 hours after infection using a Millicel-ERS2 Volt-Ohm Meter (Fisher Scientific). Data were normalized to the resistance of vehicle-treated cells.

### Tight junction protein localization assays

Caco-2 cells were plated in 96-well collagen IV–coated plates at 1.5 × 10^4^ cells and cultured for 48 hours. Cells were treated with 250 μM indomethacin or vehicle control (DMSO) for 16 hours overnight. The next day, cells were intoxicated with recombinant TcdB at a concentration of 50 nM for 4 hours. After infection, cells were washed twice with PBS and fixed by incubation with 4% paraformaldehyde for 20 min. Cells were washed with PBS and permeabilized with 0.1% Triton X-100 (MP Biomedicals) for 15 min. To block nonspecific binding, cells were incubated with 10% goat serum (Fisher Scientific) for an hour at room temperature, followed by staining with primary anti–ZO-1 mouse monoclonal antibody (BD Biosciences) at a concentration of 25 mg/ml overnight at 4°C. The next day, cells were washed with 1% goat serum twice for 10 min, followed by incubation with secondary anti-mouse Alexa Fluor 488 antibody (Thermo Fisher Scientific) at a concentration of 8 μg/ml for 2 hours at room temperature. Cells were washed twice with 1% goat serum, followed by staining with 4′,6-diamidino-2-phenylindole (DAPI; Fisher Scientific) at a concentration of 300 ng/ml for 10 min at room temperature. Cells were imaged in an Evos FL Auto using the green fluorescent protein and DAPI filters at a ×20 magnification.

### RNA extraction and rt-qPCR

Caco-2 cells were plated in 24-well plates at 1.5 × 10^5^ cells per well and cultured for 48 hours. Cells were treated with 250 μM indomethacin or vehicle control (DMSO) overnight for 16 hours. The next day, cells were intoxicated with 50 nM recombinant TcdB for 4 hours. After treatment, RNA was harvested using the RNeasy Mini Kit (Qiagen). The harvested RNA was quantified and normalized to 200 ng. Complementary DNA was synthesized from 200 ng of RNA using M-MLV Reverse Transcriptase (Promega) following the manufacturer’s instructions.

Human primer pairs were purchased from Integrated DNA Technologies (IDT). The primer sequences used were as follows: *il1b*, TGATGGCCCTAAACAGATGAAG (forward) and ATCCAGAGGGCAGAGGTCC (reverse); *cxcl8*, ATGACTTCCAAGCTGGCCGT (forward) and TCCTTGGCAAAACTGCACCT (reverse); *il10*, GGTTGCCAAGCCTTGTCTGA (forward) and AGGGAGTTCACATGCGCCT (reverse); *tjp1*, CAACATACAGTGACGCTTCACA (forward) and CACTATTGACGTTTCCCCACTC (reverse); *ocln*, ACAAGCGGTTTTATCCAGAGTC (forward) and GTCATCCACAGGCGAAGTTAAT (reverse); and *gapdh*, TGTAGACCATGTAGTTGAGGTCA (forward) and AGGTCGGTGTGAACGGATTTG (reverse). Primers were used at a 50 μM concentration. rt-qPCR was performed using iQ SYBR Green Supermix (Bio-Rad) in a reaction volume of 10 μl with 2 μl of sample and ran on the CFX384 Touch Real Time Detection System (BioRad). Cycling conditions were followed as indicated by the iQ SYBR Green Supermix manufacturer protocol. Data were normalized to the housekeeping gene *gapdh* and plotted as relative gene expression.

### PGE_2_ measurements

Caco-2 cells were plated in 24-well plates at 1.5 × 10^5^ cells per well and cultured for 48 hours. Cells were treated with 250 μM indomethacin or vehicle control (DMSO) overnight for 16 hours. The next day, cells were intoxicated with 50 nM recombinant TcdB for 4 hours. After treatment, levels of PGE_2_ were measured in cell culture supernatants using the Prostaglandin E_2_ ELISA Kit–Monoclonal (Cayman) following the manufacturer’s instructions.

### Flow cytometry analyses in CECs

To test cell death mechanisms, Caco-2 cells were plated in 24-well plates at 1.5 × 10^5^ cells per well and cultured for 48 hours. Cells were treated with 250 μM indomethacin or vehicle control (DMSO) overnight for 16 hours. The next day, cells were intoxicated with 50 nM recombinant TcdB for 4 hours. Cells were treated with 3% paraformaldehyde for 30 min as a positive control. After treatment, cells were detached from the plate by treatment with TrypLE Express (Thermo Fisher Scientific) and incubation for 5 min at 37°C. Next, cells were washed twice with staining buffer (2% FBS in PBS). Cells were stained with Pacific Blue annexin V antibody (BioLegend) and propidium iodide (BioLegend) in annexin V binding buffer (BioLegend) following the manufacturer’s instructions. Cells were washed once and resuspended in annexin V binding buffer. Cells were immediately acquired in a Cytek Aurora. To identify apoptotic cells, cells were gated on annexin V–positive propidium iodide–negative cells. For necrotic cells, cells were gated on annexin V–positive propidium iodide–positive cells.

To assess mitochondrial functions, Caco-2 cells were plated in 24-well plates at 1.5 × 10^5^ cells per well and cultured for 48 hours. Cells were treated with 250 μM indomethacin, 2 mM R2PPA, or vehicle control (DMSO) overnight for 16 hours. The next day, cells were intoxicated with recombinant TcdB at a concentration of 50 nM or treated with 2 μM carbonyl cyanide m-chlorophenylhydrazone (CCCP) as a positive control both for 4 hours. After treatment, cells were detached from the plate by treatment with TrypLE Express and incubation for 5 min at 37°C. Cells were washed twice with staining buffer (2% FBS in PBS). Cells were stained with 50 μM Mitotracker Green (Thermo Fisher Scientific) and 50 μM Mitotracker Deep Red (Thermo Fisher Scientific), 2.5 μM MitoSox (Thermo Fisher Scientific), 100 nM TMRM (Thermo Fisher Scientific), and LIVE/DEAD Fixable Aqua (Thermo Fisher Scientific) at a final dilution of 1:1000 and incubated for 30 min at 37°C. After this, cells were washed once and resuspended in staining buffer. Cells were immediately acquired in a Cytek Aurora. Mitochondrial dyes were gated on live cells, and populations of damaged mitochondria were identified as previously reported (*44*).

### Murine epithelial cell isolation

Mice were sacrificed on day 3, and colons were collected and opened longitudinally. Feces were removed, fats were trimmed, and tissues were washed in cold PBS until clean. Colons were collected in 10 ml cold 0.5% BSA in PBS on ice until all animals were processed. Tissues were then transferred into a conical with a stir bar and 10 ml of prewarmed 1× Hanks’ balanced salt solution with 1 mM EDTA, 10 mM Hepes, and 1 mM dithiothreitol. Tissues were stirred at 150 rpm for 10 min at 37°C in a heated water bath. After 10 min, tubes were shaken gently for 20 s. Tissues were removed, and cell supernatants were spun down at 500*g* and 4°C for 5 min. Supernatants were removed, and cell pellets were resuspended in 2 ml of TrypLE Express. Cells were incubated for 10 min at 25°C, and cells were pipetted up and down every 5 min during incubation. After 10 min, 10 ml of cold PBS were added, and cells were passed through a 70-μm cell strainer. Cells were spun down at 500*g* and 4°C for 5 min. After this, cells were resuspended in staining buffer and counted with Trypan Blue. Cells were stained for 30 min at 37°C with 50 μM Mitotracker Green and 50 μM Mitotracker Deep Red, 2.5 μM MitoSox, 100 nM TMRM, anti-mouse CD45 antibody (BioLegend), anti-mouse CD326 (EpCAM) antibody (Thermo Fisher Scientific), and LIVE/DEAD Fixable Aqua. After this, cells were washed once and resuspended in staining buffer. Cells were immediately acquired in a Cytek Aurora. Mitochondrial dyes were gated on live CD45-negative, EpCAM-positive cells.

## Notes

View/request a protocol for this paper from *Bio-protocol*.
