# Supplementary material for: Nonsteroidal anti-inflammatory drugs sensitize epithelial cells to Clostridioides difficile toxin–mediated mitochondrial damage
Source: Sci Adv. 2023 Jul 19;9(29):eadh5552. doi: 10.1126/sciadv.adh5552 (PMC10355836; doi:10.1126/sciadv.adh5552)
Supplement: Supplementary file 1 — Figs. S1 and S2 [file sciadv.adh5552_sm.pdf]

Science Advances
AAAS

Supplementary Materials for

Nonsteroidal anti-inflammatory drugs sensitize epithelial cells to *Clostridioides difficile* toxin–mediated mitochondrial damage

Joshua Soto Ocaña *et al.*

Corresponding author: Joseph P. Zackular, joseph.zackular@pennmedicine.upenn.edu

Sci. Adv. **9**, eadh5552 (2023)
DOI: 10.1126/sciadv.adh5552

This PDF file includes:

Figs. S1 and S2

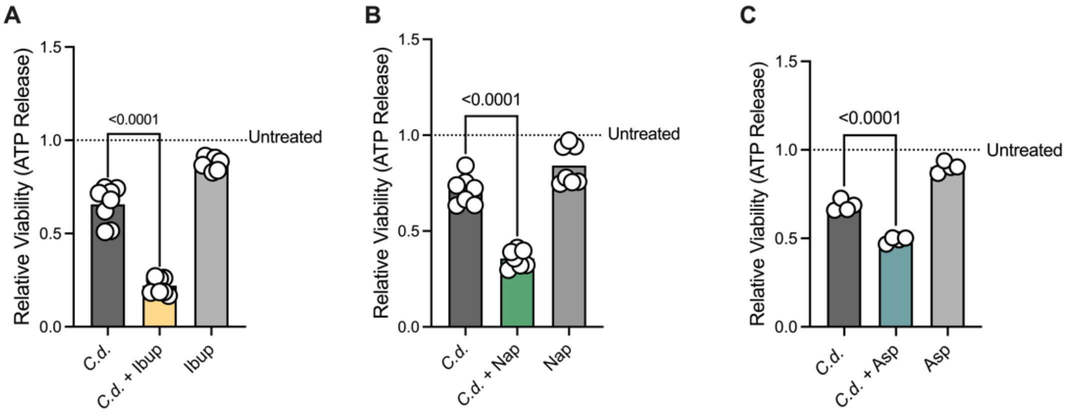

Figure S1. NSAID-mediated cell death is not limited to indomethacin
(**A,B,C**) Epithelial cell viability measured after treatment with ibuprofen (ibup), naproxen (nap), aspirin (asp), intoxication with filtered sterilized *C. difficile* (*C.d.*) supernatants at a 1:5 dilution, combination of *C. difficile* and ibuprofen (*C.d.* + ibup), *C. difficile* and naproxen (*C.d.* + nap), *C. difficile* and aspirin (*C.d.* + nap), vehicle control or mock infected. Data was normalized to mock infected and vehicle control treated cells (Untreated dotted line) (mean, n=8 or 4 per treatment, one-way ANOVA with multiple comparisons).

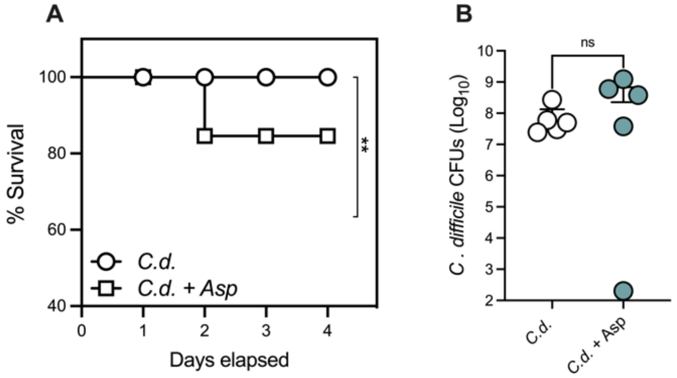

Figure S2. NSAID-induced mortality is not limited to indomethacin
Mice pre-treated with aspirin followed by infection with *C. difficile* Cd196 (*C.d.*) were (**A**) monitored for survival (n=5 mice per group, log-rank test). (**B**) *C. difficile* burdens from stool of infected mice (n=5 mice per group, non-parametric t-test).
